# Viral community acquired pneumonia at the emergency department: Report from the pre COVID‐19 age

**DOI:** 10.1002/jmv.26980

**Published:** 2021-04-06

**Authors:** Ornella Spagnolello, Alessandra Pierangeli, Maria Civita Cedrone, Valentina Di Biagio, Massimo Gentile, Annalisa Leonardi, Camilla Valeriano, Giuseppe Pietro Innocenti, Letizia Santinelli, Cristian Borrazzo, Alessandro Russo, Giuseppe Oliveto, Agnese Viscido, Massimo Ciccozzi, Giuliano Bertazzoni, Gabriella d'Ettorre, Giancarlo Ceccarelli

**Affiliations:** ^1^ Department of Public Health and Infectious Diseases University of Rome Sapienza Rome Italy; ^2^ Emergency Department University of Rome Sapienza Rome Italy; ^3^ Laboratory of Virology, Department of Molecular Medicine University of Rome Sapienza Rome Italy; ^4^ Department of Public Health and Infectious Diseases University of Pisa Pisa Italy; ^5^ Unit of Medical Statistics and Molecular Epidemiology University Campus Bio‐Medico of Rome Rome Italy

**Keywords:** community acquired pneumonia, emergency department, public health, viral infection

## Abstract

The role of viruses in community acquired pneumonia (CAP) has been largely underestimated in the pre‐coronavirus disease 2019 age. However, during flu seasonal early identification of viral infection in CAP is crucial to guide treatment and in‐hospital management. Though recommended, the routine use of nasopharyngeal swab (NPS) to detect viral infection has been poorly scaled‐up, especially in the emergency department (ED). This study sought to assess the prevalence and associated clinical outcomes of viral infections in patients with CAP during peak flu season. In this retrospective, observational study adults presenting at the ED of our hospital (Rome, Italy) with CAP from January 15th to February 22th, 2019 were enrolled. Each patient was tested on admission with Influenza rapid test and real time multiplex assay. Seventy five consecutive patients were enrolled. 30.7% (*n* = 23) tested positive for viral infection. Of these, 52.1% (*n* = 12) were H1N1/FluA. 10 patients had multiple virus co‐infections. CAP with viral infection did not differ for any demographic, clinic and laboratory features by the exception of CCI and CURB‐65. All intra‐ED deaths and mechanical ventilations were recorded among CAP with viral infection. Testing only patients with CURB‐65 score ≥2, 10 out of 12 cases of H1N1/FluA would have been detected saving up to 40% tests. Viral infection occurred in one‐third of CAP during flu seasonal peak 2019. Since not otherwise distinguishable, NPS is so far the only reliable mean to identify CAP with viral infection. Testing only patients with moderate/severe CAP significantly minimize the number of tests.

## Highlights

This study stresses the incidence of viral pneumonia during the flu season and underlies the role of viral infection screening at the ED regardless of the current COVID‐19 pandemic.

## 1 INTRODUCTION

The role of viruses in lower respiratory tract illness has been long time underestimated especially in the emergency setting, where the priority is generally to focus on identifying critical patients and to rapidly start empirical antimicrobial therapy.^1^

However, influenza‐like illness (ILI) pathogens and seasonal influenza viruses causes significant morbidity and mortality worldwide each year, and their identification in patients admitted with community acquired pneumonia (CAP) has multiple implications on in‐hospital patient management.^2^, ^3^ Moreover, it has been postulated that intercurrent viral respiratory infections are able to modulate ACE2 receptors leading to upper airway mucosal damage and local immune impairment.^3^, ^4^ Therefore, ILI (mainly caused by influenza viruses, parainfluenza virus and respiratory syncytial virus) could represent a predisposing factor for subsequent severe acute respiratory syndrome coronavirus 2 (SARS‐CoV‐2) infection.^4^, ^5^ Finally, the identification of the causal pathogen of CAP is even more crucial now that the clinical and radiological differential diagnosis between coronavirus diasease 2019 (COVID‐19) and no‐SARS‐CoV‐2 viral pneumonia is controversial.^6^

For this reason, we reviewed the cases of CAP admitted at the emergency department (ED) of a large university hospital in a period of epidemiological peak for ILI and influenza. The primary aim of our study was to assess the prevalence of viral infections in patients with CAP during 2019 ILI and flu season's peak. The secondary aims were to investigate (a) intra‐ED clinical outcomes of CAP testing positive for viral etiologies (intra‐ED death, intensive care units [ICU] admission) and (b) the impact of targeted versus nontargeted screening for viral infections.

## 2 METHODS

### 2.1 Study design

This is a retrospective, single center, observational study involving patients with CAP attending the adult ED of a 1200‐bed in‐town teaching Hospital (Rome, Italy) with a catchment area of 600,000‐1,200,000 people.^7^

The study period was set between January 15th and February 22th, 2019, during the peak of 2018–19 flu season. The Italian nationwide sentinel surveillance network (InfluNet) reported for 2018–19 influenza season: (1) 8 million cases of ILI in Italy (incidence of 13.6%), (2) a more significant co‐circulation of influenza A(H1N1)pdm09 and A(H3N2) virus subtypes, (3) an influenza vaccination coverage in elderly population equal to 53.1%.^8^

### 2.2 Eligibility criteria and definitions

All adult (>18 years) admitted at the ED with a definitive diagnosis of CAP were selected and included in the study. Patients admitted to hospital for 48 h or more in the 90 days before this presentation were excluded.^9^ CAP definition was compliant with current American Thoracic Society and Infectious Diseases Society of America guideline.^10^

### 2.3 Data collection

Demographic, anamnestic and clinical data, laboratory results (routine bloods analyses, including baseline arterial blood gas and C‐reactive protein), and radiological features (chest X‐Ray/computrd tomography scan) along with pneumonia severity scores (CURB‐65 and qSOFA) were obtained from ED patients' files. Nasopharyngeal swab (NPS) test results collected and analyzed at the time of ED hospitalization were obtained from the virology laboratory database.

### 2.4 Microbiologic molecular assays

Microbiological tests consisted of Influenza rapid test and Multiplex real‐time PCR Assay. NPS samples were collected by trained staff within 4 h from ED admission; biological samples were freshly tested for influenza virus A and B with a rapid molecular test (Xpert Xpress Flu; Cepheid), providing results in 30–40 min. An aliquot from each NPS was first stored at −80°C and then processed in batch using a Multiplex real‐time PCR Assay (FTD respiratory pathogens 21 plus; Fast Track Diagnostics), for detection of a panel of 17 viruses and 5 bacteria [influenza A/A‐H1N1/B; rhinovirus (HRV); coronaviruses NL63, 229E, OC43, HKU1; parainfluenza (PIV) 1‐4; metapneumovirus; bocavirus (HBOV); respiratory syncytial virus (RSV); adenovirus (ADV); enterovirus; parechovirus; *Mycoplasma pneumoniae; Chlamydophila pneumoniae; Staphylococcus aureus; Streptococcus pneumoniae; Haemophilus influenzae*].

Bacteriological examination of the sputum samples was not available for all patients and therefore was not considered in this study.

### 2.5 Statistical analysis

For categorical variables, either Pearson's *χ* ^2^ or Fisher's exact test were used to test the statistical difference in proportion between two or three independent groups. The level of agreement between tests was determined using Cohen's *κ* coefficient. Description of median with interquartile range (25%–75%), mean and *SD*, simple frequencies (*n*), proportions and rates of the given data on each variable was calculated. All data were analyzed using Statistical Package for Social Science version 20.

### 2.6 Ethical considerations

Data exposed in this study were previously collected for diagnostic and clinical purposes by the medical staff of ED and the virology laboratory. The study was carried out in accordance with the Helsinki Declaration and data were collected and analyzed after receiving patients' informed consent. Ethical approval was not required since the study was based on data routinely collected and stored anonymized according to the Italian law on privacy.^11^

## 3 RESULTS

### 3.1 Overall study population

**Figure 1:**
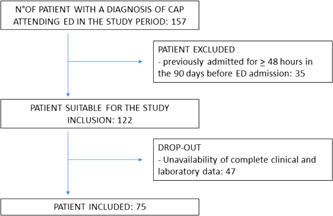
Flowchart of patient enrollment in final analysis. CAP, community acquired pneumonia; ED, emergency department

The flow chart providing an overview of patient enrollment is showed in Figure 1. Overall, out of 157 patients with CAP presenting at the ED during the study period, only 75 patients met eligibility criteria and were eventually included in the study analysis. Demographic and clinical data of the study population along with outcomes are listed in Table 1.

**Table 1:** **Study population**: Demographic and clinical data along with outcomes of overall study population and groups

	**All patients**	**All viral infections**	**Flu**	
	**(*n* = 75)**	**(*n* = 23)**	**(*n* = 12)**	
Demographic	Male, *n* (%)	32 (42.6%)	10 (43.4%)	5 (41.6%)
	Age, mean (*SD*)	72.4 (17.2)	76.4 (12.3)	77.5 (10.3)
	Vaccination, *n* (%)	18 (24%)	4 (17.3%)	1 (8.3%)
	Smoke, *n* (%)	22 (29.3%)	7 (30.4%)	5 (41.6%)
	CCI	5 (3–6)	5 (3.5–5.3)	5 (4.8–6.0)
	COPD, *n* (%)	13 (17.3%)	3 (13.0%)	2 (16.6%)
Severity scores	qSOFA	0 (0–0)	1 (0–1)	1 (0.8–1)
	CURB‐65	2 (1–2.8)	2 (0.8–3)	3 (2–3.3)
Respiratory findings	O_2_ Saturation, %	97% (93%–98%)	96% (92%–97%)	98% (98%–100%)
	Respiratory rate, *n*	19 (17–22)	20 (18–25.5)	21 (18–25)
	P/F ratio	300 (264.5–344.5)	309.5 (281–405.0)	290 (162–349)
	Lactate (mmol/L)	1 (0.8–1.7)	1.3 (0.8–2.3)	1.2 (0.9–2.4)
Laboratory results	WBC (x10^9^/L)	8.6 (5.8–11.8)	9.7 (8.2–10.7)	7.4 (3.4–9.8)
	Neutrophilia, %	80.2 (71.3–85.4)	75.8 (74.3–87.9)	83.9 (75.3–86.2)
	CRP (mg/dl)	5.6 (1.4–10.5)	4.9 (0.6–8.9)	6.3 (2.59–8.0)
Radiographic findings	Interstitial pattern, *n* (%)	23 (30.6%)	8 (34.7%)	6 (50.0%)
	Consolidation(s), *n* (%)	73 (97.3%)	22 (95.6%)	11 (91.6%)
Outcomes	CPAP/NIV, *n* (%)	5 (6.6%)	3 (13.0%)	2 (16.6%)
	Mechanical ventilation, *n* (%)	2 (2.6%)	2 (8.6)	2 (16.6%)
	Death in ED, *n* (%)	1 (1.3%)	1 (4.3%)	1 (8.3%)

*Note:* Data are presented as median with CI unless otherwise stated.

Abbreviations: CCI, Charlson comorbidity index; CI, confidence interval; COPD, chronic obstructive pulmonary disease; CPAP/NIV, continuous positive airway pressure/non invasive ventilation; CRP, C‐reactive protein; WBC, white blood cells.

### 3.2 Prevalence of viral infections in patients with CAP and etiology

A total of 23/75 (30.7%) of the overall study sample was positive for viral infection. In particular, a single viral pathogen was determined in 14/75 (18.7%), two viral agents in 3/75 (4.0%), and a viral‐bacterial co‐infection in 6/75 (8.0%). A bacterial agent was detected in 19/75 NPS (25.3%) whereas 33/75 (44.0%) tested negative.

**Figure 2:**
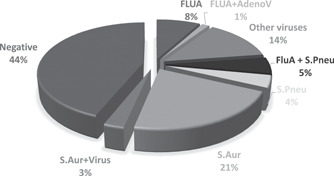
Pathogens prevalence in the study population. The number of cases and the percentage for each viral or bacterial pathogen are reported below the graph. FLU A, influenza A any subtype; S. Aur, staphylococcus aureus; S. Pneu, streptococcus pneumoniae

Influenza A was the virus more frequently detected: 12 patients resulted positive to Influenza A by the FTD assay while only 8 of these were also identified by the rapid test; the two tests had good agreement (94.7%; κ = 0.77; *p* < .001). Of the Influenza A cases, 10 were typed as H1N1 whereas the remaining two, not typed, were probably H3N2 strains. Other detected viruses were HRV (*n* = 4), RSV (*n* = 3), HBOV (*n* = 2), ADV (*n* = 2), PIV‐2 (*n* = 1), CoV 229E (*n* = 1), and HMPV (*n* = 1). Viral co‐infections were detected in a total of 3 cases, namely: Influenza A H1N1/ADV; RSV/HBOV and HRV/HBOV. *Staphylococcus aureus* was the bacterium more frequently detected (18/25) of which two were in co‐infection, one with Influenza A and the other with HRV. *Streptococcus pneumoniae* was the other bacterium detected, found in seven cases of which four were in co‐infection with Influenza A (Figure 2).

### 3.3 Laboratory results did not differ among CAP testing positive and negative for viral etiologies

As shown in Table 1, no significant differences were observed in terms of inflammatory markers levels among patients with CAP of different etiologies.

### 3.4 Intra‐ED clinical outcomes of CAP testing positive for viral etiologies

CAP with or without viral infection did not differ for any demographic, clinic and laboratory features with the exception of overall Charlson Comorbidity Index (CCI) (5 [4–6] vs. 4 [2–5.3]; *p* = .015) and CURB‐65 (3 [1.3–3] vs. 2 [0.8–2]; *p* = .011) (Table 1). 86.6% (*n* = 65) of patients were eventually admitted on ward level whereas one was directly referred to ICU; ED intra‐mortality rate was 1.3% (*n* = 1). Although not statistically relevant, all mechanical ventilation (MV) and intra‐ED deaths were recorded into the group of CAP with viral infection. Notably, the only patient who died in the ED had received mechanical ventilation before.

### 3.5 Impact of Nontargeted vs targeted testing for viral infection

**Figure 3:**
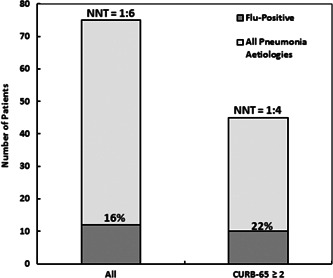
Nontargeted vs targeted screening for viral infection. This plot compares two strategies of screening patients with CAP for viral infection at the ED. Nontargeted test includes all patients presenting with CAP regardless of pneumonia severity. On the contrary, targeted screening involves only moderate to severe CAP (scoring 2 or more at CURB‐65 pneumonia severity score). CAP, community acquired pneumonia; ED, emergency department; NNT, number needed to test

Testing all patients presenting at the ED during the study period, regardless of pneumonia severity, we detected 23/75 (30.7%) CAP positive for viral infection. Of these, about half, 12/23, were Influenza A infections, either H1N1 or Flu A not‐typed. The number needed to test (NNT) for Influenza was 1:6.25 (Figure 3).

On the contrary, testing only patients scoring 2 or more at CURB‐65 pneumonia severity score (*n* = 45), the ratio of CAP positive for viral infection was 17 out of 45 (37.7%). Since 10 out of 17 were either H1N1 or Flu A not‐typed, the NNT for Influenza raised to 1:4.5.

### 3.6 Impact of universal influenza test execution on ED activity

According to internal protocol, patient waiting for influenza rapid test' results were isolated in dedicated spaces and asked to wear a surgical mask as a precautionary measure. Considering that (1) the results of rapid test were available in 30–40 min, (2) only 16% of patients (those affected by influenza) required respiratory isolation, (3) the overall median staying in the ED was 2 days (1–4), the length of staying in dedicated isolation spaces was minimized to approximately 1–5 h.

## 4 DISCUSSION

Despite traditionally underestimated, especially in the ED setting, more recent pre‐COVID‐19 evidence suggested that viruses play a relevant role in CAP etiology.^12^, ^13^, ^14^, ^15^, ^16^ The Etiology of Pneumonia in the Community study was a large prospective US based surveillance study in which one or more viruses were detected in 26% of CAP requiring hospitalization.^14^ In our study out of 75 CAP presenting at the ED during the peak of the flu season 2018/2019, around 30% tested positive for viral infection and, of these, approximately 50% were either H1N1 or Flu A not‐typed. This number keeps up with previously reported data despite our observation was limited to the Influenza season's peak.

Despite many efforts aiming at validating clinical scores able to discriminate patients with Influenza, no syndromic formula has shown to be performant enough to support physicians in their decision making.^17^, ^18^, ^19^, ^20^ Accordingly, no significant differences were found in our study from the comparison of CAP with or without viral infection and with or without Influenza. Therefore, NPS molecular testing is so far the only reliable mean to detect viral infections.

Although not statistically significant, in our study all intra‐ED deaths and MV were recorded into the group of CAP with viral infection. This data is in line with previous evidence underling the relation among viral infection and pneumonia severity.

Furthermore, taking into consideration the economic concerns around nontargeted screening, we sought the hypothesis to test for viral infection only moderate to severe CAP bound to be admitted. Interestingly, targeting the screening to this group of patients we detected 10 out 12 Influenza cases saving 40% of overall tests. This result complies with the assumption that viral pneumonias (mostly related to Influenza in the pre‐COVID‐19 era) tend to be more severe than other causes of pneumonia and stresses the importance of these patients to be tested.

Saving as much as 40% of molecular tests may be way more feasible in a contest of shortness of supply, as in the middle of a pandemic. However, the value of this observation is limited to the rationalization and maximization of the yield of viral diagnostic tests in terms of ability to intercept true positive patients. In fact, untested patients remain with a dubious etiology and therefore empirical therapy must be based on epidemiological, clinical and etiological findings and must in any case consider both bacterial and viral etiology as much possible.

Finally, testing patients presenting at the ED with CAP especially during the influenza season's peak is crucial both for therapy optimization and for in‐hospital infection control. Viral identification in CAP ensures appropriate antiviral treatments and reduces the deployment of unnecessary antibiotic therapy. Moreover, in our experience we believe that prompt identification of patients with influenza not only minimized aerosol transmission during the ED staying but also ensured a better allocation of infected patients on ward level limiting intra‐hospital transmission.

This study has several limitations principally related to retrospective design and single center enrollment. The small sample size affected the statistical power of our observations and prevented us from performing advanced analysis. However, it offers a real‐life glimpse of a major ED during a flu epidemic. Hence, our results need to be replicated by larger prospective multicenter studies. If confirmed, our findings might support viral infection screening at the ED for patients presenting with CAP during influenza season peak regardless of a concomitant pandemic.

## 5 CONCLUSION

This study enlightens the key role of viral infection screening on CAP during influenza season peak even in the pre COVID‐19 age.^1^, ^12^, ^13^, ^14^, ^15^, ^16^, ^21^, ^22^ Although largely underestimated especially in the ED setting, it is well reckoned that testing patient with CAP for viral infection offers multiple advantages ranging from compliance with antibiotic stewardship to prevention of in‐hospital outbreak. Since differences in clinical presentation are not relevant, a quick and sensitive molecular diagnosis could improve patients' triage in EDs.

## CONFLICT OF INTERESTS

The authors declare that there are no conflict of interests.

## AUTHOR CONTRIBUTIONS

Ornella Spagnolello and Giancarlo Ceccarelli. conceived the study design, interpreted data and wrote the manuscript. Maria Civita Cedrone, Valentina Di Biagio, Camilla Valeriano, and Annalisa Leonardi were responsible of patient recruitment and collecting data at the ED. Alessandra Pierangeli, Massimo Gentile, Giuseppe Oliveto, Agnese Viscido were on charge for virologic laboratory analysis. Cristian Borrazzo was responsible of data analysis. Alessandra Pierangeli, Giuseppe Pietro Innocenti, and Letizia Santinelli participated in writing the manuscript. Ornella Spagnolello, Giancarlo Ceccarelli., Alessandra Pierangeli, Gabriella d'Ettorre, Massimo Ciccozzi, Giuliano Bertazzoni, and Alessandro Russo were responsible of critical revision of the manuscript for intellectual content.

## Data Availability

Unpublished data are available by email request from the corresponding author.
